# Association of Blood Plasma Vascular Endothelial Growth Factor and Testicular Artery Doppler Parameters with Canine Sperm Freezability

**DOI:** 10.3390/ani16162500

**Published:** 2026-08-11

**Authors:** Florin Petrișor Posastiuc, Guillaume Domain, Lotte Spanoghe, Joke Lannoo, Penelope Banchi, Mircea Petcu, Ann Van Soom

**Affiliations:** 1 Department of Internal Medicine, Reproduction and Population Medicine, Faculty of Veterinary Medicine, Ghent University, Salisburylaan 133, 9820 Merelbeke, Belgium; guillaume.domain@ugent.be (G.D.); lotte.spanoghe@ugent.be (L.S.); joke.lannoo@ugent.be (J.L.); ann.vansoom@ugent.be (A.V.S.); 2 Department of Clinical Sciences II, Faculty of Veterinary Medicine, University of Agronomic Sciences and Veterinary Medicine of Bucharest, 105 Blvd. Splaiul Independentei, 050097 Bucharest, Romania; 3 Department of Animal Breeding & Genomics, Wageningen University & Research, Droevendaalsesteeg 1, 6708 PB Wageningen, The Netherlands; penelopebanchi@gmail.com; 4 Department of Paraclinical Sciences, Faculty of Veterinary Medicine, University of Agronomic Sciences and Veterinary Medicine of Bucharest, 105 Blvd. Splaiul Independentei, 050097 Bucharest, Romania; mircea-mihai.petcu@doctorat.usamv.ro

**Keywords:** canine semen, sperm cryopreservation, sperm freezability, vascular endothelial growth factor, testicular artery Doppler, sperm motility, sperm kinematics

## Abstract

Semen cryopreservation is widely used in canine breeding, but individual variation in post-thaw semen quality limits its efficiency. This study investigated whether testicular artery Doppler-derived blood-flow parameters and circulating vascular endothelial growth factor (VEGF) concentrations are associated with semen quality and sperm freezability in clinically healthy dogs. Sixty-four privately owned male dogs underwent reproductive screening, semen collection, testicular Doppler ultrasonography, blood sampling, fresh and post-thaw semen analysis, and plasma VEGF quantification. Testicular artery Doppler parameters were not significantly associated with fresh or post-thaw semen quality or with most freezability outcomes. Plasma VEGF concentration was positively correlated with fresh linearity (LIN) (*p* = 0.034), straightness (STR) (*p* = 0.031), and beat-cross frequency (BCF) (*p* = 0.043). Higher VEGF concentrations were associated with greater post-thaw declines in progressive motility (PM) (*p* = 0.036), straight-line velocity (VSL) (*p* = 0.032), LIN (*p* = 0.001), STR (*p* = 0.005), and wobble (WOB) (*p* = 0.010). In multivariable analysis, VEGF was associated with linearity decline (B = 0.595, 95% CI: 0.254–0.937; *p* = 0.001). Circulating VEGF may therefore be associated with individual variation in canine sperm cryotolerance, whereas testicular artery Doppler assessment showed limited value.

## Simple Summary

Freezing dog semen is commonly used in breeding because it allows valuable genetic material to be stored and transported. However, semen quality after thawing can vary greatly between dogs, making it difficult to predict how well a sample will tolerate freezing. This study examined whether testicular artery blood flow and the blood levels of vascular endothelial growth factor, a protein involved in blood vessel function, were related to semen quality and freezing tolerance in healthy dogs. Sixty-four privately owned male dogs were examined. Each dog underwent reproductive evaluation, semen collection, testicular ultrasound, blood sampling, and semen assessment before and after freezing. Testicular blood-flow measurements were not clearly related to semen quality or to how well sperm tolerated freezing. In contrast, higher blood levels of vascular endothelial growth factor were associated with greater decreases in selected sperm movement characteristics after thawing, especially the ability of sperm cells to move in a straight and efficient direction. These findings suggest that vascular endothelial growth factor may help identify dogs whose semen is more likely to be sensitive to freezing.

## 1. Introduction

Semen cryopreservation is a cornerstone assisted reproductive technology in canine breeding, enabling long-term storage of genetic material and supporting artificial insemination programs, including the use of semen from geographically distant males [1,2]. However, sperm cryotolerance, the capacity of spermatozoa to withstand the cooling, osmotic, and oxidative stress of freezing and thawing, varies markedly among individual dogs, even when standardized protocols are applied [3,4]. Reliable predictors of this individual variation, termed sperm freezability, remain poorly established [4,5,6].

Spermatogenesis is a highly regulated process that depends on the structural and functional integrity of the testis, as well as an adequate and stable blood supply [7]. Testicular perfusion is primarily provided by the testicular artery, which forms a convoluted supratesticular segment prior to entering the testis [8]. Because testicular blood flow has limited autoregulatory capacity, even subtle alterations in vascular supply may adversely affect spermatogenesis [8,9].

Doppler ultrasonography offers a non-invasive method to assess testicular artery blood flow, providing both qualitative and quantitative information on these blood flow characteristics [10]. Parameters such as peak systolic velocity (PSV), end-diastolic velocity (EDV), resistive index (RI), and pulsatility index (PI) have been associated with sperm quality in dogs [11,12,13]. However, whether these Doppler-derived parameters can predict sperm freezability in dogs remains unknown.

In addition to hemodynamic factors, molecular regulators of vascular function may also reflect testicular physiology and, consequently, sperm quality and cryoresilience. Vascular endothelial growth factor (VEGF) is a key mediator of angiogenesis and vascular permeability, playing a central role in the regulation of blood vessel formation and function [14,15]. Because spermatogenesis is highly dependent on adequate testicular perfusion, local vascular insufficiency or hypoxia may trigger a compensatory upregulation of VEGF via the hypoxia-inducible factor 1-alpha (HIF-1α) pathway, the principal transcriptional mechanism driving VEGF expression under hypoxic conditions [16]. As VEGF produced locally can diffuse into the systemic circulation, circulating VEGF concentration has previously been investigated as an indicator of underlying vascular pathology in dogs, most notably in hemangiosarcoma, and in inflammatory conditions [14,17]. By analogy, circulating VEGF concentration may similarly serve as an indirect, systemic indicator of underlying testicular vascular stress, complementing the direct hemodynamic information provided by Doppler ultrasonography. However, the role of VEGF in male reproduction remains less clearly defined, with limited evidence suggesting an association with several aspects of male fertility, including sperm motility, sperm survival, and testicular function [18,19].

The aim of the present study was to investigate whether testicular artery Doppler-derived blood-flow parameters and circulating VEGF concentrations are associated with semen quality and sperm freezability in clinically healthy dogs. Specifically, we evaluated the relationships between Doppler parameters, blood plasma VEGF levels, fresh and post-thaw sperm quality, and the magnitude of cryopreservation-induced decline in selected sperm functional parameters.

## 2. Materials and Methods

### 2.1. Study Design and Experimental Workflow

This prospective observational study included privately owned male dogs presented to the Small Animal Teaching Hospital of Ghent University between February 2025 and June 2025. Ethical approval was granted by the Ethical Committee for Animal Experiments of the Faculty of Veterinary Medicine and the Faculty of Bioscience Engineering at Ghent University (protocol code: 2024-087; approval date: February 2025).

**Figure 1:**
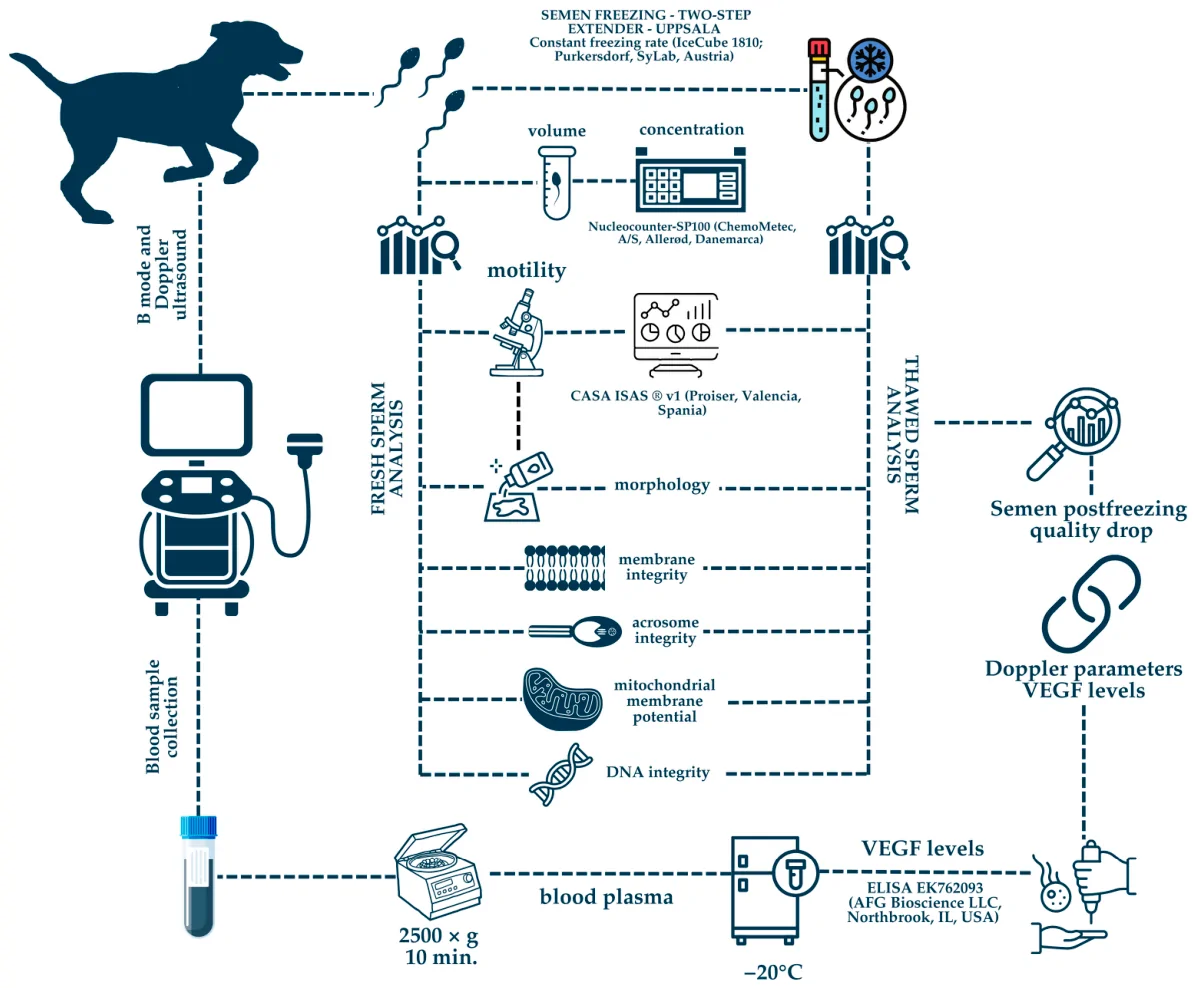
Schematic overview of the study design and analytical workflow. The diagram summarizes the sequence of ultrasonographic assessment, semen collection and cryopreservation, blood sampling, fresh and post-thaw semen quality evaluation, blood plasma VEGF quantification, and assessment of cryopreservation-induced changes in semen quality. VEGF, vascular endothelial growth factor; ELISA, enzyme-linked immunosorbent assay; CASA, computer-assisted sperm analysis.

The study protocol included standardized reproductive screening to determine eligibility according to the criteria listed in Table 1, followed by semen collection and cryopreservation, blood collection, testicular and prostatic B-mode and Doppler ultrasonography, fresh and post-thaw semen quality evaluation and blood plasma VEGF levels quantification. Moreover, a minimum 5-day abstinence period for each dog was confirmed by the owners before admission. A schematic overview of the study design is presented in Figure 1.

To avoid potential stress-related alterations in ejaculate characteristics, semen was collected before any other procedure. Following collection, dogs were walked and allowed to recover before Doppler ultrasonography, which was performed only after sexual arousal and penile erection were no longer evident, approximately 30 min after ejaculation. Clinical examination and ultrasonographic assessment were subsequently completed, whilst macroscopical examination of the ejaculate was done immediately after the collection. Dogs in which any of these assessments revealed findings compatible with the predefined exclusion criteria were excluded from the final study population.

### 2.2. Semen Collection and Cryopreservation

Semen collection, fresh semen handling and cryopreservation were performed according to a previously described protocol [4]. Briefly, the second, sperm-rich fraction and the third, prostatic fraction were collected by digital manipulation into sterile plastic vials and maintained at 37 °C until analysis. The third fraction was assessed macroscopically only. The volume of the sperm-rich fraction was recorded, after which sperm concentration was determined using a NucleoCounter^®^ SP-100 system (ChemoMetec A/S, Allerød, Denmark). Total sperm output (TSO) was calculated as sperm concentration multiplied by the volume of the sperm-rich fraction. Immediately thereafter, an aliquot of the sperm-rich fraction was processed for further sperm analyses, while the remaining sample was cryopreserved.

Sperm cryopreservation was carried out using an adapted two-step Uppsala method [23], based on the protocol described by Domain et al. (2022) [24]. Briefly, seminal plasma was removed by centrifugation at 720× *g* for 5 min at 22 °C. The sperm pellet was sequentially diluted in TRIS–citric acid–fructose extenders containing egg yolk, glycerol and Equex STM paste, with a 2 h equilibration period at 5 °C between dilution steps. Final sperm concentration before freezing was adjusted to 100–200 × 10^6^ spermatozoa/mL. Samples were loaded into 0.5 mL straws, frozen using a programmable freezing device (IceCube 1810, SyLab, Purkersdorf, Austria) according to Schäfer-Somi et al. (2006), and stored in liquid nitrogen at −196 °C [25].

### 2.3. Sperm Analysis

Fresh and frozen–thawed semen samples were subjected to standard computer-assisted sperm analysis (CASA) for the assessment of motility and velocity parameters. Sperm morphology was evaluated using eosin–nigrosin staining. Plasma membrane integrity, acrosome integrity, and mitochondrial membrane potential were assessed using fluorescence-based assays, whereas DNA integrity was evaluated using toluidine blue staining. Cryopreserved straws were thawed individually in a 37 °C water bath for 30 s prior to evaluation.

#### 2.3.1. CASA Evaluation and Morphology

For CASA, an ISAS^®^v1 system was used (Proiser, Valencia, Spain). Samples were first diluted in prewarmed TRIS–citric acid–fructose medium (37 °C) to obtain a final concentration of 30–60 × 10^6^ spermatozoa/mL. Thereafter, 4.5 µL of each diluted sample was loaded into an ISAS^®^D4C20 chamber (Proiser, Valencia, Spain), and four microscopic fields were recorded for analysis. Acquisition settings were based on Domain et al. (2022), with the following modifications: 45 consecutive frames were captured at 60 frames/s, particle size detection was restricted to 10–80 µm^2^, and spermatozoa with a curvilinear velocity (VCL) < 20 µm/s were classified as immotile [26]. The following endpoints were obtained: total motility (TM, %), progressive motility (PM, %), average path velocity (VAP, µm/s), straight-line velocity (VSL, µm/s), VCL (µm/s), amplitude of lateral head displacement (ALH, µm), beat-cross frequency (BCF, Hz), wobble (WOB, %), straightness (STR, %), and linearity (LIN, %).

Eosin–nigrosin-stained smears were used for sperm morphology assessment under bright-field microscopy at 1000× magnification (Olympus BX51TF, Tokyo, Japan). A total of 200 spermatozoa were evaluated per sample and further classified into minor and major defects according to a previous approach [4].

#### 2.3.2. Fluorescence-Based Assays

All fluorescence-based assessments were performed using a Leica DMR epifluorescence microscope (Leica Microsystems, Wetzlar, Germany) equipped with a mercury lamp and appropriate filter sets. For each assay, 200 spermatozoa were evaluated per sample at 1000× magnification.

Plasma membrane integrity was evaluated using SYBR-14/propidium iodide (PI) staining, adapted from Domain et al. (2022) [24]. Semen was diluted in HEPES-TALP to 25 × 10^6^ spermatozoa/mL. SYBR-14 and PI were added at final concentrations of 100 nM and 12 µM, respectively, with each staining step followed by incubation for 5 min at 37 °C in the dark. After staining, 10 µL of the suspension was placed on a glass slide and covered with a coverslip. Spermatozoa showing green head fluorescence were classified as membrane-intact, whereas those showing red head fluorescence were classified as membrane-damaged.

Acrosome integrity was assessed using fluorescein isothiocyanate-conjugated peanut agglutinin staining (FITC-PNA; Sigma-Aldrich, Steinheim, Germany), according to a protocol adapted from Almadaly et al. (2012) [27]. Semen was diluted in HEPES-TALP to 25 × 10^6^ spermatozoa/mL, with a final volume of 250 µL. A 10 µL aliquot was smeared onto a glass slide and air-dried. The smear was fixed in absolute ethanol for 2 min, air-dried, rinsed with phosphate-buffered saline (PBS), and dried again. Slides were then incubated with FITC-PNA solution (20 µg/mL) for 30 min, rinsed with PBS, air-dried, and mounted with DABCO under a coverslip. Spermatozoa with a bright, continuous acrosomal cap were considered acrosome-intact, whereas cells with absent, incomplete, or disrupted acrosomal fluorescence were classified as acrosome-damaged.

Mitochondrial membrane potential was evaluated using JC-1 staining (5,5′,6,6′-tetrachloro-1,1′,3,3′-tetraethylbenzimidazolylcarbocyanine iodide; Molecular Probes, Eugene, OR, USA), following a protocol adapted from Domain et al. (2022) [24]. Semen was diluted in HEPES-TALP to 25 × 10^6^ spermatozoa/mL, with a final volume of 250 µL. JC-1 was then added to obtain a final concentration of 36 µM, and the sample was incubated for 5 min at 37 °C in the dark. After incubation, 10 µL of the stained suspension was placed on a glass slide and covered with a coverslip. Spermatozoa showing orange fluorescence in ≥50% of the midpiece were classified as having high potential, whereas those showing orange fluorescence in <50% of the midpiece were classified as having low potential.

#### 2.3.3. Toluidine Blue Staining

DNA integrity was assessed by toluidine blue staining, following Residiwati et al. (2020) [28]. A 30 µL semen aliquot was smeared onto a glass slide, air-dried, fixed in freshly prepared 96% ethanol–acetone solution (1:1, *v*/*v*) at 4 °C for 30 min, and air-dried again. The smear was then hydrolyzed in 0.1 N HCl at 4 °C for 5 min, air-dried, and stained with 30 µL of 0.05% toluidine blue in McIlvain citrate–phosphate buffer (pH 3.5) for 1 h at room temperature. After drying, spermatozoa with light-blue heads were considered DNA-intact, while those with dark-blue to purple heads were classified as DNA-damaged.

### 2.4. Doppler Assessment

With each dog maintained in a standing position, the testicular artery was identified bilaterally by color Doppler ultrasonography using a MyLab™50 XVision ultrasound system (Esaote, Genova, Italy) equipped with a microconvex transducer operating at 5.0–9.0 MHz. The transducer was positioned at the scrotal level, near the base of the testis and close to the cranial pole, to allow visualization of the looped segment of the distal supratesticular artery, as previously described by Zelli et al. (2013) [13]. After vessel identification, pulsed-wave Doppler mode was activated, and the sample volume was positioned in the center of the arterial lumen. For each artery, three consecutive Doppler recordings were obtained, with each recording including at least three complete, clearly distinguishable cardiac cycles.

Ultrasound settings were standardized according to Venianaki et al. (2023) [29]. The Doppler wall filter was set at 100 Hz to minimize tissue-motion artifacts while preserving physiologically relevant blood-flow signals. The insonation angle was maintained at 40° ± 5°, within the accepted range of 0–60°, to reduce errors in velocity estimation. Color gain was adjusted to minimize background noise and was maintained at approximately 64%. The pulse repetition frequency was set between 0.7 and 1.4 kHz.

Five hemodynamic parameters were recorded: peak systolic velocity (PSV, cm/s), end-diastolic velocity (EDV, cm/s), systolic/diastolic ratio (S/D), resistive index (RI), and pulsatility index (PI). The S/D ratio was calculated as PSV/EDV, the RI was calculated as (PSV − EDV)/PSV, and the PI was calculated as (PSV − EDV)/TAMV, where TAMV represents the time-averaged maximum velocity. For each artery, the mean of the three repeated measurements was calculated. Thereafter, bilateral mean values were obtained for each dog and used for statistical analysis.

### 2.5. Assessment of Plasma VEGF Levels

Venous blood samples were collected into lithium heparin tubes (BD Vacutainer, Plymouth, UK). Following collection, samples were centrifuged at 2500× *g* for 10 min to obtain plasma, which was aliquoted and stored at −20 °C until analysis. Blood samples were collected from conscious dogs during the same clinical visit as semen collection; time of day, feeding status, exercise, and acclimation period prior to sampling were not standardized.

Blood plasma VEGF concentrations were measured in singlicate using a commercially available canine sandwich enzyme-linked immunosorbent assay (ELISA) kit (AFG Bioscience LLC, EK762093, Northbrook, IL, USA), according to the manufacturer’s instructions. Standards were prepared by serial dilution using the provided diluent, with the reagent blank serving as the zero-concentration. Standards were analysed in duplicate on the supplied 96-well microplate. For sample wells, 40 µL of sample diluent and 10 µL of plasma were added per well. The plate was gently mixed, sealed, and incubated at 37 °C for 40 min. After incubation, the wells were aspirated and washed five times with 300 µL of wash buffer per well. Subsequently, 50 µL of horseradish peroxidase (HRP)-conjugate reagent was added to each well, except the blank well, and the plate was incubated at 37 °C for 40 min. Following a second washing step, color development was performed by adding 50 µL each of Chromogen Solution A and Chromogen Solution B. The plate was protected from light and incubated at 37 °C for 20 min, after which the reaction was stopped by adding 50 µL of Stop Solution to each well. Optical density was measured at 450 nm using a microplate reader within 15 min of reaction termination (Multiskan™ GO spectrophotometer, Thermo Fisher Scientific, Vantaa, Finland). VEGF concentrations were calculated from the standard curve generated from duplicate standards. The assay had an analytical sensitivity of 0.78 pg/mL and a detection range of 6.25–200 pg/mL, with manufacturer-reported intra-assay and inter-assay coefficients of variation of <8% and <10%, respectively. None of the VEGF values obtained in this study fell outside the assay’s detection range.

### 2.6. Statistical Analysis

Sperm freezability was defined as the change between fresh and post-thaw values for the evaluated sperm parameters. For statistical modelling and correlation analysis, freezability outcomes were expressed as the absolute post-thaw decline, calculated as the difference between fresh and post-thaw values, with higher values indicating a greater reduction after thawing. Relative post-thaw variation was calculated separately as [(post-thaw − fresh)/fresh] × 100 and used for graphical visualization of individual responses to cryopreservation and for descriptive identification of the endpoints showing the greatest proportional decrease. Data were analysed using IBM SPSS Statistics version 26.0 (IBM Corp., Armonk, NY, USA). Normality was evaluated using the Shapiro–Wilk test (α = 0.05), and due to non-normal distributions, data are reported as median and interquartile range (IQR), with the 25th and 75th percentiles. Differences between fresh and post-thaw sperm parameters were assessed using the Wilcoxon signed-rank test.

Associations between circulating VEGF concentrations, Doppler parameters and sperm freezability variables were initially screened using Spearman rank correlation coefficients. Multivariable linear regression models were constructed to evaluate whether VEGF and testicular vascular parameters were associated with the decline in sperm quality following cryopreservation. Associated factor selection was guided by biological plausibility and evidence of association.

Pre-model data screening was performed to identify potential extreme values. Outliers were identified using median absolute deviation (MAD)–based standardized scores, with deviations from the median scaled by a factor of 1.4826. Values with an absolute MAD-based z-score > 3.5 were flagged and visually inspected.

Given the physiological interdependence among Doppler variables derived from systolic and diastolic velocities (PSV, EDV, PI, RI and S/D), collinearity was assessed through Spearman correlation coefficients. Doppler variables with ρ > 0.85 were considered collinear and were not entered simultaneously into the same model.

Model-specific influence diagnostics were assessed in the final regression models using Cook’s distance, leverage values, studentized residuals and Mahalanobis distance. High leverage was defined as values exceeding 2(k + 1)/*n*, where k is the number of predictors and *n* the sample size. Multivariate outliers were identified using a χ^2^ threshold corresponding to *p* < 0.001 for Mahalanobis distance, and extreme residuals were defined as |studentized residual| > 3. No observations exceeded accepted thresholds, indicating that model estimates were not driven by influential observations. Linearity and homoscedasticity of each regression model were assessed by plotting standardized residuals against standardized predicted values. Normality of residuals was assessed using a normal probability (P-P) plot and the Shapiro–Wilk test.

To assess the representativeness of the subset of dogs subjected to fluorescence-based assays and DNA integrity testing, this subgroup was compared with the remaining dogs for age, body weight, size class (small ≤ 10 kg, medium 10–25 kg, large ≥ 25 kg [30], used as a proxy for breed distribution given the high breed heterogeneity of the study population), baseline semen parameters, sperm kinematics, normal morphology, and plasma VEGF concentration, using the Mann–Whitney U test for continuous variables and the Chi-square test for size class.

Absolute post-thaw decline was used as the primary freezability metric. To evaluate the potential influence of fresh baseline values on this metric, key associations were additionally assessed using the relative percentage post-thaw decline as a sensitivity analysis.

## 3. Results

A total of 64 dogs representing 33 different breeds were included in the study. The median age was 3.75 years (IQR: 2.09–6.06), and the median body weight was 29.4 kg (IQR: 18.45–36.63).

Each dog underwent semen collection only once during the study period. The median volume of the sperm-rich fraction was 1.5 mL (IQR: 1.00–2.28), with a median sperm concentration of 332.25 × 10^6^ spermatozoa/mL (IQR: 206.30–485.78). The median TSO was 433.11 × 10^6^ spermatozoa/ejaculate (IQR: 276.42–948.46).

All semen samples were subjected to CASA and sperm morphology evaluation. In addition, all 64 dogs underwent Doppler ultrasonographic assessment and plasma VEGF measurement. Fluorescence-based assays and DNA integrity assessment were performed on a subset of 33 samples. This subset was defined by sample availability rather than random selection. The subset of 33 dogs did not differ significantly from the remaining 31 dogs in age, body weight, size class, fresh semen volume, sperm concentration, TSO, fresh motility and kinematic parameters, normal morphology, or plasma VEGF concentration (all *p* > 0.05), with the exception of BCF, which showed a borderline difference (*p* = 0.049). Given the number of comparisons performed, this isolated finding is consistent with a chance result and does not suggest a systematic difference between groups, supporting the representativeness of the subset for the fluorescence-based and DNA integrity assessments (Table S1). A sensitivity power analysis (two-tailed, α = 0.05, power = 0.80) indicated that, with the subset sample size of 33, correlations of |r| ≥ 0.466 or greater can be reliably detected.

Screening of the final dataset for outliers identified six flagged observations, including one dog with extreme values concerning two different variables. As none of these observations were associated with measurement or data-entry errors and all were deemed biologically plausible, no data were excluded.

### 3.1. Semen Quality and Post-Freezing Variation

Median values and interquartile ranges for the evaluated motility and kinematic parameters in fresh and post-thaw semen samples are shown in Table 2. A significant decrease was observed in all motility and kinematic parameters after thawing (*p* ≤ 0.001), except for ALH, which did not differ significantly (*p* = 0.323).

Normal sperm morphology had a median value of 79.0% (IQR: 66.75–85.38) in fresh samples and decreased significantly to 72.75% (IQR: 58.38–78.38; *p* ≤ 0.001) after freezing. Minor defects increased from a median of 10.25% (IQR: 6.63–18.38) in fresh samples to 15.50% (IQR: 10.63–22.88; *p* ≤ 0.001) after freezing, while major defects increased from 14.50% (IQR: 9.63–19.75) to 18.00% (IQR: 14.13–23.25; *p* ≤ 0.001).

Plasma membrane integrity decreased significantly from 86% (IQR: 81–89.5) in fresh samples to 67% (IQR: 59.5–74.5) after thawing (*p* ≤ 0.001). Similarly, acrosome integrity declined from 88% (IQR: 80.5–91) to 71% (IQR: 64–78; *p* ≤ 0.001), while mitochondrial membrane potential showed a marked reduction from 81% (IQR: 76–86) to 4% (IQR: 1–11; *p* ≤ 0.001). DNA integrity exhibited a smaller but still significant decrease, from 98% (IQR: 96–98) in fresh samples to 97% (IQR: 96–98) post-thaw (*p* = 0.014).

**Figure 2:**
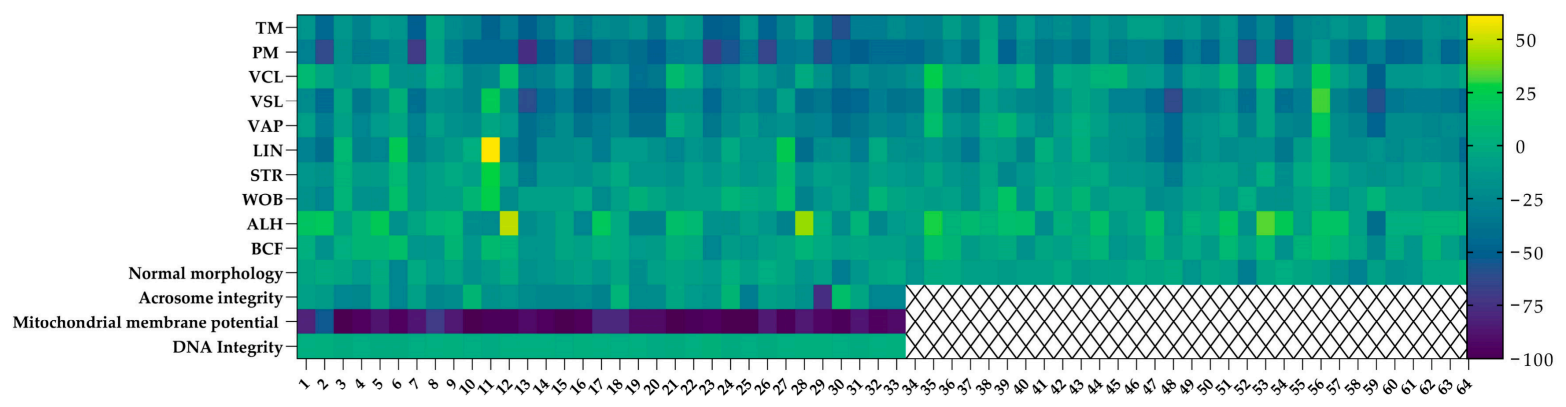
Relative post-thaw changes in semen quality endpoints for individual dogs. Values were calculated for each dog as [(post-thaw − fresh)/fresh] × 100. Rows represent semen quality endpoints, and columns represent individual dogs (1–64). For plasma membrane integrity, acrosome integrity, DNA integrity, and mitochondrial membrane potential, data were available for 33 samples only. Negative values indicate a post-thaw decrease, values close to zero indicate minimal change, and positive values indicate an increase after thawing. The color scale represents the magnitude and direction of relative change (%). TM, total motility; PM, progressive motility; VCL, curvilinear velocity; VSL, straight-line velocity; VAP, average path velocity; WOB, wobble; STR, straightness; LIN, linearity; ALH, amplitude of lateral head displacement; BCF, beat-cross frequency.

Relative post-thaw changes were calculated to compare the magnitude of cryopreservation-induced decline across semen quality endpoints. The greatest median reductions were observed in mitochondrial membrane potential (95.5%; IQR: 98.76–86.6) and PM (43.08%; IQR: 54.22–31.89) whilst the median relative variation in DNA integrity bordered 0% (IQR: 2.01–0). The distribution of relative changes across individual dogs is illustrated in Figure 2, highlighting marked inter-individual variability in sperm freezability.

### 3.2. Doppler Parameters

Median and IQR values for all Doppler parameters are presented in Table 3.

PSV was significantly correlated with the age of the dogs (ρ = 0.260, *p* = 0.038), while EDV values were correlated with weight (ρ = 0.295, *p* = 0.018), as were RI (ρ = −0.251, *p* = 0.045) and S/D (ρ = −0.249, *p* = 0.038). Regarding baseline ejaculate characteristics (volume, concentration, TSO), the only relevant correlation was observed between EDV and TSO (ρ = 0.299, *p* = 0.016).

Furthermore, as shown in Table 4, no significant correlations were observed between the recorded Doppler parameters and any motility, velocity, or morphology parameters in either fresh or post-thaw semen samples (all *p* > 0.05).

Moreover, no significant correlations were identified between any Doppler parameter and plasma membrane integrity, acrosome integrity, mitochondrial membrane potential, or DNA integrity, in either fresh or post-thaw semen samples (all *p* > 0.05) (Table 5).

### 3.3. Blood Plasma VEGF Levels

Median VEGF levels were 19.29 pg/mL (IQR: 16.76–25.18), with no significant correlation with either age or body weight of the dogs (all *p* > 0.05). Moreover, no significant correlation was identified between VEGF levels and any of the recorded Doppler parameters or baseline ejaculate characteristics, including volume, concentration, and TSO (all *p* > 0.05).

VEGF levels showed weak but significant positive correlations with LIN (ρ = 0.265, *p* = 0.034), STR (ρ = 0.270, *p* = 0.031), and BCF (ρ = 0.254, *p* = 0.043) in fresh semen. No significant correlations were observed between VEGF levels and any motility or kinematic parameter in post-thaw semen samples (all *p* > 0.05). Moreover, no significant correlations were found between VEGF levels and sperm morphology in either fresh or post-thaw samples (all *p* > 0.05). Similarly, no significant correlations were identified between VEGF levels and acrosome integrity, DNA integrity, or mitochondrial membrane potential in either fresh or post-thaw samples (all *p* > 0.05). The only significant correlation observed in post-thaw parameters was between VEGF levels and membrane integrity (ρ = −0.404, *p* = 0.020).

### 3.4. VEGF, Doppler and Freezability

**Figure 3:**
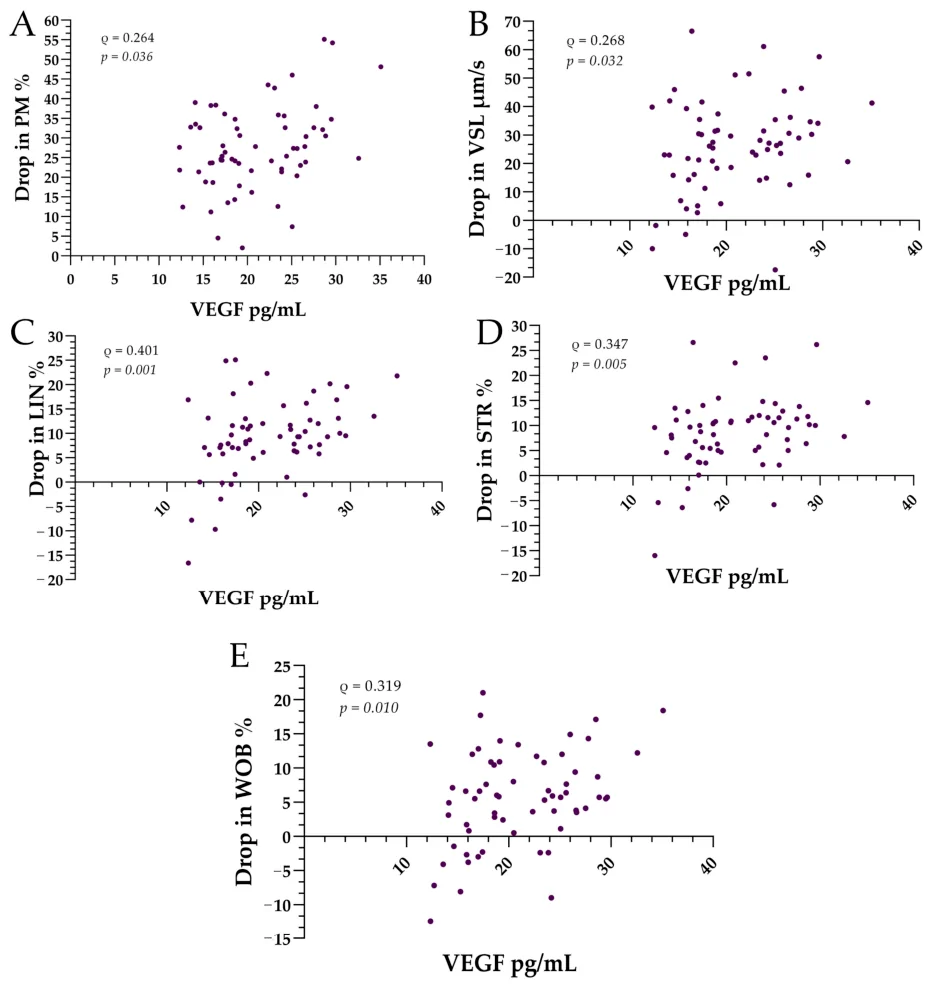
Association between blood plasma VEGF and post-thaw decreases in selected sperm motility and kinematic parameters. Scatterplots show the relationship between VEGF (pg/mL) and the drop after freezing in PM (**A**), VSL (**B**), LIN (**C**), STR (**D**), and WOB (**E**). Spearman’s correlation coefficient (ρ) and corresponding *p*-values are reported within each panel. VEGF, vascular endothelial growth factor; PM, progressive motility; VSL, straight-line velocity; LIN, linearity; STR, straightness; WOB, wobble.

Assessment of the magnitude of cryopreservation-induced semen quality loss showed that none of the recorded Doppler parameters were significantly associated with the extent of reduction in motility, velocity or morphology parameters (*p* > 0.05). In contrast, blood plasma VEGF concentrations were significantly correlated with the magnitude of decrease in PM (ρ = 0.264, *p* = 0.036), VSL (ρ = 0.268, *p* = 0.032), LIN (ρ = 0.401, *p* = 0.001), STR (ρ = 0.347, *p* = 0.005) and WOB (ρ = 0.319, *p* = 0.010) (Figure 3). 

Regarding the decrease in plasma membrane integrity, acrosome integrity, mitochondrial membrane potential, and DNA integrity after thawing, neither the Doppler parameters nor blood plasma VEGF levels showed any significant correlations (*p* > 0.05) (Table S2).

Following correlation analysis, exploratory multivariable regression models were built for the freezability outcomes significantly associated with circulating VEGF concentrations (Table 6). These models aimed to assess the combined explanatory value of VEGF and selected testicular Doppler parameters for cryopreservation-induced sperm quality decline. Because several motility and kinematic variables were interrelated, the decline in LIN was selected as the primary modelled outcome because it showed the strongest univariable association with VEGF. Following the predefined collinearity assessment, RI was selected as the representative Doppler factor for the main model because of its biological relevance as an index of intratesticular vascular resistance.

The primary multivariable regression model, including blood plasma VEGF concentration, RI, age, and body weight as explanatory variables, was statistically significant (*p* = 0.009) and explained 20.1% of the variability in LIN decline. Blood plasma VEGF concentration was the only significant independent associated factor with LIN decline (B = 0.595, 95% CI: 0.254–0.937; β = 0.413; *p* = 0.001), indicating that higher blood plasma VEGF concentrations were associated with a greater post-thaw reduction in LIN.

Secondary multivariable regression models were then fitted for the remaining freezability outcomes that showed significant univariable associations with blood plasma VEGF concentration. In the model using PM decline as the dependent variable, the overall model did not reach statistical significance, although a non-significant trend was observed (*p* = 0.083). Within this model, blood plasma VEGF concentration was significantly associated with PM decline (B = 0.681, 95% CI: 0.192–1.170; β = 0.345; *p* = 0.007), indicating that higher blood plasma VEGF concentrations were associated with a greater post-thaw decrease in PM.

For VSL decline, the overall model was not statistically significant (*p* = 0.341), and blood plasma VEGF concentration showed only a positive but non-significant association with the outcome (B = 0.741, 95% CI: −0.030 to 1.511; β = 0.246; *p* = 0.059). In the model using STR decline as the dependent variable, the overall model did not reach statistical significance, although a non-significant trend was observed (*p* = 0.093). Blood plasma VEGF concentration was significantly associated with STR decline (B = 0.434, 95% CI: 0.106–0.762; β = 0.328; *p* = 0.010), indicating that higher blood plasma VEGF concentrations were associated with a greater post-thaw decrease in STR.

The secondary model using WOB decline as the dependent variable was statistically significant (*p* = 0.010). In this model, blood plasma VEGF concentration was significantly associated with WOB decline (B = 0.484, 95% CI: 0.182–0.787; β = 0.379; *p* = 0.002). RI was also significantly associated with WOB decline (B = 21.386, 95% CI: 3.539–39.233; β = 0.298; *p* = 0.020), suggesting that higher intratesticular vascular resistance was associated with greater WOB decline after thawing.

Across all secondary models, age and body weight were not significantly associated with any of the evaluated outcomes. No relevant multicollinearity was observed among the analysed factors. Residual diagnostics supported the assumptions of linearity, homoscedasticity, and normality for the primary model (LIN decline; Shapiro–Wilk W = 0.968, *p* = 0.099), and the same diagnostic procedure was applied to the four secondary models.

To evaluate whether these associations were sensitive to the choice of cryotolerance metric, key correlations between VEGF and post-thaw decline were repeated using relative percentage decline instead of absolute decline. The associations between VEGF and decline in VSL (ρ = 0.282, *p* = 0.024), LIN (ρ = 0.411, *p* = 0.001), STR (ρ = 0.323, *p* = 0.009), and WOB (ρ = 0.330, *p* = 0.008) remained statistically significant and consistent in direction with the absolute decline analysis. The association between VEGF and decline in progressive motility, significant using the absolute metric (*p* = 0.036), was attenuated using the relative metric (ρ = 0.217, *p* = 0.085). When the primary multivariable regression model was repeated using relative linearity decline as the outcome, the overall model was statistically significant (*p* = 0.008). Blood plasma VEGF concentration remained the only significant independent associated factor (B = 1.330, 95% CI: 0.593 to 2.067; β = 0.427; *p* = 0.001), consistent with the original model based on absolute LIN decline (B = 0.595, 95% CI: 0.254 to 0.937; β = 0.413; *p* = 0.001).

## 4. Discussion

This study showed that higher blood plasma VEGF levels were associated with greater post-thaw declines in selected canine sperm motility and kinematic parameters, particularly linearity. In contrast, testicular artery Doppler parameters were not significantly associated with the magnitude of post-thaw reduction in motility, velocity, morphology, or sperm integrity parameters.

Similar to pioneering studies, such as that of Zelli et al. (2013), Doppler evaluation of the testicular artery revealed waveforms characteristic of a low-resistance vessel [13]. These were characterized by a monophasic blood flow pattern, with slow systolic flow followed by a prolonged diastolic decrease and a relatively high end-diastolic velocity. Moreover, the median values of the monitored Doppler parameters, including PSV, EDV, PI, RI, and S/D, were close to those previously reported in healthy dogs by Zelli et al. (2013) [13] and Venianaki et al. (2023) [29], thereby supporting the validity of the Doppler measurements obtained in the present study.

Interestingly, previous reports have suggested that certain Doppler parameters may be associated with fresh semen quality in dogs [13]. In particular, RI and PI were negatively correlated with total and progressive motility at different time points after semen collection, suggesting that higher vascular resistance may be associated with poorer sperm motility [13]. These parameters were also negatively associated with the percentage of membrane-intact spermatozoa, whereas EDV showed a positive correlation with this parameter [13]. However, this relationship was not confirmed in the present study, as none of the evaluated Doppler parameters showed significant correlations with fresh semen motility, kinematic, or morphology parameters. This discrepancy may be partly explained by methodological differences between studies. In the study by Zelli et al. (2013) [13], semen evaluation relied on conventional methods, including subjective motility assessment by phase-contrast microscopy and the hypo-osmotic swelling test for plasma membrane integrity. Moreover, as the semen samples in that study originated from only five dogs, the reported correlations may have been influenced by individual animal effects or study-specific trends. In contrast, the present study included a larger population of 64 dogs and applied more objective and standardized semen evaluation methods, which may have reduced observer-dependent variability and provided a more robust assessment of semen quality endpoints.

Similarly, differences in other sperm quality parameters, such as DNA integrity, may also contribute to divergent findings among studies. Lemos et al. (2020) reported a negative correlation between EDV and sperm oxidative DNA damage using a formamidopyrimidine-DNA glycosylase (FPG)-modified comet assay [12]. FPG is a DNA repair enzyme that recognizes oxidized purine bases, thereby allowing the comet assay to specifically detect oxidative DNA lesions [31]. In contrast, the present study assessed DNA integrity using toluidine blue staining, which primarily reflects sperm chromatin integrity or condensation status rather than oxidative DNA damage [32]. This may explain why no association was observed between the recorded Doppler parameters and nuclear integrity in the present study. The present findings should not be interpreted as a contradiction to those of Lemos et al. (2020), but rather as reflecting the fact that oxidative DNA damage and chromatin integrity represent related but distinct aspects of sperm nuclear quality [12].

In contrast to testicular artery Doppler parameters, VEGF has been studied primarily in pathological contexts, including tumor development and inflammatory processes, whereas its potential relevance to dog reproductive function remains uncharacterized [17,33,34]. Queiroz et al. (2014) reported circulating VEGF concentrations in blood plasma from healthy dogs, with mean values of 11.7 ± 16.4 pg/mL [35]. In the present study population, the median plasma VEGF concentration was 19.29 pg/mL (IQR: 16.76–25.18), which was broadly comparable to previously reported values. This agreement suggests that circulating VEGF concentrations in the dogs included in the present study were within the expected range for clinically healthy animals.

In fresh semen, blood plasma VEGF concentration showed weak but significant positive correlations with LIN, STR, and BCF. Although these associations were modest, they may suggest a link between systemic VEGF status and selected aspects of sperm movement pattern under basal, non-cryopreserved conditions. Interestingly, evidence from human sperm studies supports a potential relationship between VEGF and sperm motion characteristics [18]. In vitro exposure of human spermatozoa to VEGF has been reported to improve selected motility parameters in a concentration-dependent manner, with the greatest effect observed at 15 ng/mL, where motility, progression, straight-line velocity, and curvilinear velocity were significantly increased [18].

Higher plasma VEGF concentrations were correlated with greater post-thaw decreases in PM, VSL, LIN, STR, and WOB. The exploratory regression models added further context to these correlations by evaluating whether the association between VEGF and sperm freezability persisted when Doppler parameters, age, and body weight were considered simultaneously. The strongest support was observed for LIN decline, for which the overall model was significant and VEGF remained the only independent associated factor. Overall, these findings indicate that VEGF may be linked not only to fresh sperm motility characteristics, but also to how these are preserved under cryogenic conditions. A possible explanation for these findings is that circulating VEGF may reflect the vascular and stress-related status of the male reproductive system. VEGF is involved in angiogenesis, vascular permeability, and tissue oxygenation, and is expressed in the testis and epididymis [36]. Thus, increased VEGF levels may occur as a compensatory response to local hypoxia, inflammation, oxidative stress, or altered vascular function. These conditions may negatively affect the testicular and epididymal environment in which spermatozoa are produced and mature. As a result, sperm cells may acquire subtle defects in membrane composition, mitochondrial function, or oxidative balance, which may not always be evident in fresh semen evaluation but can reduce their ability to withstand the thermal, osmotic, and oxidative stress imposed by cryopreservation [3].

From a more mechanistic perspective, circulating VEGF may reflect a systemically elevated redox/oxidative signaling tone, partly because VEGF signaling itself is known to act through NADPH oxidase-derived reactive oxygen species (ROS) [37]. Since low, physiological ROS levels are required for normal sperm motility and capacitation-like membrane changes [38], this could plausibly explain the positive association observed between VEGF and fresh LIN, STR, and BCF. However, spermatozoa operate within a narrow redox window: their plasma membranes are rich in polyunsaturated fatty acids and their cytoplasmic antioxidant and repair capacity is limited, making them highly susceptible to oxidative damage once ROS exceeds this physiological range [39]. Cryopreservation is itself a well-established independent trigger of ROS generation and depletes antioxidant defenses, imposing an additional oxidative burden on spermatozoa [39]. We therefore hypothesize that dogs with higher circulating VEGF, potentially reflecting a higher baseline oxidative tone, may have spermatozoa that function normally, or even optimally, under fresh conditions, but that carry reduced antioxidant reserve. This reduced reserve would not be apparent during fresh evaluation but would leave these cells less able to buffer the additional oxidative insult of freeze–thaw, resulting in greater post-thaw functional decline. This redox-based hypothesis is, to our knowledge, not directly tested in canine sperm and is proposed here based on established VEGF and sperm redox biology rather than on direct mechanistic evidence from the present study.

Previous studies have shown that altered VEGF signaling can affect male reproductive function [19,36]. In transgenic mice, VEGF overexpression in the testis and epididymis was associated with infertility and spermatogenic arrest, suggesting that VEGF may act not only on endothelial cells but also on non-endothelial target cells within the male reproductive tract [19]. However, this interpretation remains speculative. Further studies assessing VEGF expression and VEGF receptor distribution in the testis, epididymis, seminal plasma, and mature spermatozoa are required to determine whether VEGF directly contributes to spermatogenic or post-testicular processes that influence sperm cryotolerance.

From a clinical perspective, the association with LIN decline and the non-significant trend observed for PM decline may be relevant because these parameters describe different aspects of effective sperm movement. While PM reflects the proportion of spermatozoa moving progressively, LIN indicates more specifically the directional efficiency of their trajectory. Thus, a post-thaw reduction in LIN may suggest that spermatozoa retain motility but exhibit less efficient forward movement. This is clinically important because insemination doses for frozen semen are commonly adjusted according to post-thaw progressivity, which remains a key parameter in breeding management [40].

On the other hand, Doppler parameters did not show a consistent association with sperm freezability in the present study. None of the recorded Doppler variables were significantly correlated with the cryopreservation-induced decline in motility, velocity, morphology, or sperm functional integrity parameters, with the exception of RI in the WOB regression model. This may indicate that a single resting assessment of testicular blood flow does not fully reflect the intrinsic sperm characteristics that determine cryotolerance. Moreover, Doppler ultrasonography reflects vascular conditions at the time of examination, whereas the spermatozoa evaluated after ejaculation developed over several weeks before sampling [41]. Repeated Doppler assessments over time, across a spermatogenic cycle, may provide a more representative evaluation of testicular haemodynamics and could better identify whether dogs with persistently different vascular profiles also differ in semen quality or post-thaw sperm resilience. This temporal mismatch may partly explain the limited predictive value of a single Doppler measurement for post-thaw semen quality decline in clinically healthy dogs.

The association between VEGF and post-thaw decline in PM was significant when expressed as absolute decline but attenuated when expressed as relative percentage decline, suggesting this particular association may be partly influenced by the fresh baseline motility value, whereas the associations between VEGF and decline in LIN, STR, and WOB remained consistent across both metrics.

Several limitations of this study should be acknowledged. Blood sampling for VEGF quantification was not standardized for time of day, feeding status, exercise, or stress/acclimation status prior to collection, which may have added variability to VEGF measurements and influenced the observed associations with semen quality and freezability outcomes. Additionally, the study population comprised 33 different breeds, with most breeds represented by only one or two dogs, precluding breed from being modeled directly as a covariate. Although body weight was included in the multivariable models and size class was used as a proxy for breed-related variability in the subset comparison, breed-associated differences in testicular size, blood flow, and reproductive physiology cannot be fully excluded as a residual confounding factor. The absence of significant associations between Doppler parameters or VEGF and the decline in acrosome integrity, mitochondrial membrane potential, or DNA integrity should be interpreted in light of the subset size (*n* = 33), which was powered to detect correlations of |r| ≥ 0.466 or greater; weaker associations may not have been detected. Finally, no correction for multiple comparisons was applied given the exploratory nature of this study; associations identified, particularly secondary or borderline findings, should therefore be interpreted with caution and considered hypothesis-generating, warranting validation in future studies with a priori-defined primary outcomes.

## 5. Conclusions

In conclusion, testicular artery Doppler parameters were not consistently associated with semen quality or sperm freezability, suggesting limited value in clinically healthy dogs. In contrast, blood plasma VEGF concentration was associated with selected fresh sperm kinematic parameters and with greater post-thaw declines in motility and sperm movement characteristics, especially LIN. These findings suggest that circulating VEGF may be linked to individual variability in canine sperm cryotolerance; however, they should be regarded as hypothesis-generating rather than establishing VEGF as a validated clinical predictor of freezability. Independent validation in larger, prospective cohorts, ideally incorporating pre-defined thresholds and cross-validation, is required before circulating VEGF could be considered for practical use in predicting individual dogs’ sperm cryotolerance.

## Figures and Tables

**Table 1:** Inclusion and exclusion criteria applied during screening and enrolment of the male dogs. * Ultrasonographic lesions included focal or diffuse changes in echogenicity, echotexture, symmetry, margins, size, or parenchymal structure; ** Prostatic measurements were performed in both longitudinal and transverse planes. Two prostatic volume estimates were calculated separately: the actual prostatic volume, using the formula described by Atalan et al. (1999) [20], and the expected prostatic volume, estimated from age and body weight according to Ruel et al. (1998) [21]. *** Prostatomegaly was defined as an actual prostatic volume exceeding the expected prostatic volume [22].

Criteria	Inclusion	Exclusion
Age	≥12 months	<12 months
Body weight	≥10 kg	<10 kg
Body condition score	4/9–5/9	<4/9 or >5/9
Clinical examination (including examination of the testes and penis)	Clinically healthy dogs with no abnormalities detected on general clinical examination	Dogs with systemic disease or abnormalities detected on clinical examination
Testicular and prostatic B-mode ultrasound	No ultrasonographic evidence of testicular or prostatic lesions *	Presence of testicular or prostatic lesions *
Prostatic volume **	Normal prostatic volume ***	Prostatomegaly ***
Macroscopic evaluation of the ejaculate	Normal macroscopic appearance of both sperm rich and prostatic fractions	Discoloration or contamination suggestive of blood or urine

**Table 2:** Medians and interquartile ranges (IQRs: 25th–75th percentiles) for motility and kinematic parameters in fresh and post-thaw samples (*n* = 64). TM, total motility; PM, progressive motility; VCL, curvilinear velocity; VSL, straight-line velocity; VAP, average path velocity; WOB, wobble; STR, straightness; LIN, linearity; ALH, amplitude of lateral head displacement; BCF, beat-cross frequency. Asterisks indicate values significantly different from fresh semen (*p* ≤ 0.001).

	**TM %**	**PM %**	**VCL µm/s**	**VSL µm/s**	**VAP µm/s**
	Median (IQR:25th–75th Percentiles)				
Fresh	85.35 (79.4–88.48)	74 (51.75–78.28)	180.82 (163.03–198.93)	88.24 (80–100.68)	119.1 (107.67–127.33)
Post-thaw	65 * (54.95–72.06)	44.05 * (37.23–51.85)	159.5 * (136.33–175.58)	62.95 * (53.4–71.97)	95.55 * (79.45–105.2)
	**WOB %**	**STR %**	**LIN %**	**ALH µm**	**BCF Hz**
	Median (IQR:25th–75th percentiles)				
Fresh	62.88 (58.85–70.1)	76.4 (72.85–79.28)	48.7 (44.7–54.55)	3.2 (2.9–3.6)	20.4 (19.23–21.78)
Post-thaw	57.51 * (55.05–60.76)	67.79 * (64.19–71.23)	39.05 * (34.9–43.55)	3.2 (2.8–3.53)	19.4 * (18.38–21.35)

**Table 3:** Values are presented as median and interquartile range (IQR; 25th–75th percentile), (*n* = 64). PSV, peak systolic velocity; EDV, end-diastolic velocity; PI, pulsatility index; RI, resistive index; S/D, systolic/diastolic ratio.

	PSV (cm/s)	EDV (cm/s)	PI	RI	S/D
Median	14.23	7.23	0.68	0.48	1.93
IQR (25th–75th)	11.49–16.8	5.77–8.86	0.55–0.84	0.41–0.55	1.75–2.21

**Table 4:** Correlations between testicular artery Doppler parameters and semen quality parameters in fresh and post-thaw semen samples (*n* = 64). Values represent Spearman’s rank correlation coefficients, with corresponding *p*-values shown below each coefficient. TM, total motility; PM, progressive motility; VCL, curvilinear velocity; VSL, straight-line velocity; VAP, average path velocity; LIN, linearity; STR, straightness; WOB, wobble; ALH, amplitude of lateral head displacement; BCF, beat-cross frequency; PSV, peak systolic velocity; EDV, end-diastolic velocity; PI, pulsatility index; RI, resistive index; S/D, systolic/diastolic ratio.

**Fresh Semen**											
	**TM**	**PM**	**VCL**	**VSL**	**VAP**	**LIN**	**STR**	**WOB**	**ALH**	**BCF**	**Normal Morphology**
**PSV**	−0.034	0.101	−0.201	0.118	−0.019	0.190	0.201	0.194	−0.219	0.069	0.061
*p*	0.789	0.428	0.110	0.352	0.882	0.133	0.111	0.124	0.082	0.586	0.634
**EDV**	0.088	0.174	−0.032	0.021	−0.058	0.047	0.075	−0.006	−0.079	−0.025	0.011
*p*	0.487	0.169	0.802	0.869	0.652	0.710	0.555	0.962	0.533	0.844	0.928
**PI**	−0.143	−0.140	−0.195	0.028	−0.042	0.128	0.126	0.197	−0.138	0.155	0.027
*p*	0.259	0.269	0.123	0.825	0.742	0.315	0.322	0.118	0.276	0.222	0.834
**RI**	−0.160	−0.166	−0.180	0.011	−0.043	0.108	0.102	0.186	−0.122	0.145	0.013
*p*	0.208	0.190	0.155	0.931	0.736	0.394	0.423	0.141	0.337	0.254	0.916
**S/D**	−0.160	−0.187	−0.161	0.010	−0.025	0.097	0.062	0.178	−0.109	0.104	−0.001
*p*	0.205	0.140	0.204	0.937	0.842	0.448	0.625	0.159	0.392	0.413	0.993
**Post thaw semen**											
	**TM**	**PM**	**VCL**	**VSL**	**VAP**	**LIN**	**STR**	**WOB**	**ALH**	**BCF**	**Normal Morphology**
**PSV**	0.127	0.111	−0.169	−0.038	−0.101	0.025	0.030	0.051	−0.128	0.040	0.103
*p*	0.318	0.381	0.182	0.764	0.429	0.842	0.813	0.690	0.313	0.753	0.419
**EDV**	0.091	0.122	−0.093	0.005	−0.036	0.039	0.055	−0.003	−0.077	0.043	0.084
*p*	0.472	0.338	0.465	0.971	0.779	0.759	0.663	0.981	0.543	0.735	0.510
**PI**	0.043	−0.008	0.014	−0.022	−0.027	−0.047	−0.017	−0.015	0.059	0.066	0.029
*p*	0.736	0.950	0.915	0.865	0.833	0.714	0.892	0.905	0.643	0.604	0.823
**RI**	0.026	−0.029	0.021	−0.031	−0.029	−0.057	−0.036	−0.028	0.069	0.061	0.009
*p*	0.840	0.818	0.872	0.808	0.819	0.657	0.779	0.828	0.590	0.635	0.941
**S/D**	0.005	−0.047	0.036	−0.033	−0.019	−0.068	−0.051	−0.029	0.080	0.050	−0.008
*p*	0.970	0.714	0.780	0.793	0.881	0.593	0.689	0.818	0.532	0.694	0.947

**Table 5:** Correlations between testicular artery Doppler parameters and plasma membrane integrity, acrosome integrity, mitochondrial membrane potential, and DNA integrity in fresh and post-thaw semen samples (*n* = 33). Values represent Spearman’s rank correlation coefficients, with corresponding *p*-values shown below each coefficient. Doppler parameters included peak systolic velocity (PSV), end-diastolic velocity (EDV), pulsatility index (PI), resistive index (RI), and systolic/diastolic ratio (S/D).

**Fresh Semen**				
	**Plasma** **Membrane** **Integrity**	**Acrosome** **Integrity**	**Mitochondrial** **Membrane** **Potential**	**DNA** **Integrity**
**PSV**	−0.136	−0.181	−0.141	0.050
*p*	0.450	0.314	0.435	0.783
**EDV**	−0.098	0.008	0.004	−0.009
*p*	0.587	0.964	0.982	0.961
**PI**	−0.033	−0.081	−0.144	−0.110
*p*	0.856	0.654	0.423	0.543
**RI**	−0.047	−0.099	−0.174	−0.088
*p*	0.794	0.582	0.332	0.627
**S/D**	−0.009	−0.086	−0.141	−0.094
*p*	0.958	0.634	0.435	0.602
**Post-thaw semen**				
	**Plasma** **membrane** **integrity**	**Acrosome** **integrity**	**Mitochondrial** **membrane** **potential**	**DNA** **integrity**
**PSV**	0.097	0.027	0.096	−0.183
*p*	0.590	0.883	0.596	0.308
**EDV**	0.118	0.016	0.002	−0.070
*p*	0.512	0.928	0.991	0.699
**PI**	0.026	0.041	0.185	−0.060
*p*	0.886	0.823	0.303	0.740
**RI**	0.037	0.036	0.176	−0.052
*p*	0.839	0.842	0.327	0.773
**S/D**	0.024	0.008	0.173	−0.070
*p*	0.894	0.966	0.336	0.698

**Table 6:** Multivariable linear regression models evaluating the association of blood plasma VEGF concentration, RI, age, and body weight with post-thaw declines in selected sperm motility and kinematic parameters. Model *p*-values refer to the overall regression model (*n* = 64). B, unstandardized regression coefficient; β, standardized regression coefficient; CI, confidence interval; VEGF, vascular endothelial growth factor; RI, resistive index; BW, body weight; LIN, linearity; PM, progressive motility; VSL, straight-line velocity; STR, straightness; WOB, wobble.

Outcome	Model *p*-Value	R^2^	Adjusted R^2^	Associated Factor	B	95% CI	β	*p*-Value
LIN decline	0.009	0.201	0.147	VEGF	0.595	0.254 to 0.937	0.413	0.001
				RI	19.153	−0.975 to 39.281	0.236	0.062
				Age	−0.201	−1.014 to 0.612	−0.061	0.622
				BW	0.038	−0.102 to 0.178	0.066	0.590
PM decline	0.083	0.128	0.069	VEGF	0.681	0.192 to 1.170	0.345	0.007
				RI	−7.788	−36.614 to 21.037	−0.070	0.591
				Age	0.103	−1.061 to 1.267	0.023	0.861
				BW	−0.012	−0.213 to 0.189	−0.015	0.905
VSL decline	0.341	0.072	0.010	VEGF	0.741	−0.030 to 1.511	0.246	0.059
				RI	8.121	−37.299 to 53.541	0.048	0.722
				Age	0.430	−1.404 to 2.264	0.062	0.641
				BW	−0.044	−0.360 to 0.272	−0.037	0.780
STR decline	0.093	0.124	0.065	VEGF	0.434	0.106 to 0.762	0.328	0.010
				RI	3.967	−15.372 to 23.306	0.053	0.683
				Age	0.216	−0.565 to 0.997	0.072	0.582
				BW	−0.023	−0.157 to 0.112	−0.043	0.738
WOB decline	0.010	0.200	0.146	VEGF	0.484	0.182 to 0.787	0.379	0.002
				RI	21.386	3.539 to 39.233	0.298	0.020
				Age	−0.389	−1.109 to 0.332	−0.133	0.285
				BW	0.069	−0.055 to 0.194	0.137	0.268

## Data Availability

All data presented in this study are available upon request from the corresponding author.

## Supplementary Materials

The following supporting information can be downloaded at: https://www.mdpi.com/article/10.3390/ani16162500/s1, Table S1: Comparison of the 33-dog subset (fluorescence-based assays and DNA integrity testing) with the remaining 31 dogs; Table S2: Spearman correlation coefficients (ρ) between blood plasma VEGF concentration, testicular artery Doppler parameters, and the post-thaw drop in plasma membrane integrity, acrosome integrity, mitochondrial mem-brane potential, and DNA integrity (n = 33); VEGF, vascular endothelial growth factor, peak systolic velocity (PSV), end-diastolic velocity (EDV), pulsatility index (PI), resistive index (RI), and systolic/diastolic ratio (S/D).

## Abbreviations

The following abbreviations are used in this manuscript:

ALH	Amplitude of lateral head displacement
BCF	Beat-cross frequency
BW	Body weight
CASA	Computer-assisted sperm analysis
CI	Confidence interval
DABCO	1,4-Diazabicyclo[2.2.2]octane
DNA	Deoxyribonucleic acid
EDV	End-diastolic velocity
ELISA	Enzyme-linked immunosorbent assay
FITC-PNA	Fluorescein isothiocyanate-conjugated peanut agglutinin
FPG	Formamidopyrimidine-DNA glycosylase
HEPES-TALP	HEPES-buffered Tyrode’s albumin lactate pyruvate medium
HRP	Horseradish peroxidase
IQR	Interquartile range
JC-1	5,5′,6,6′-Tetrachloro-1,1′,3,3′-tetraethylbenzimidazolylcarbocyanine iodide
LIN	Linearity
MAD	Median absolute deviation
PBS	Phosphate-buffered saline
PI	Pulsatility index; propidium iodide when referring to staining
PM	Progressive motility
PSV	Peak systolic velocity
RI	Resistive index
S/D	Systolic/diastolic ratio
STR	Straightness
TAMV	Time-averaged maximum velocity
TM	Total motility
TRIS	Tris(hydroxymethyl)aminomethane
TSO	Total sperm output
VAP	Average path velocity
VCL	Curvilinear velocity
VEGF	Vascular endothelial growth factor
VSL	Straight-line velocity
WOB	Wobble
