# Supplementary material for: Association of Blood Plasma Vascular Endothelial Growth Factor and Testicular Artery Doppler Parameters with Canine Sperm Freezability
Source: Animals (Basel). 2026 Aug 11;16(16):2500. doi: 10.3390/ani16162500 (PMC13508883; doi:10.3390/ani16162500)
Supplement: Supplementary file 1 [file animals-16-02500-s001.zip › animals-4456885-supplementary.pdf]

Table S1. Comparison of the 33-dog subset (fluorescence-based assays and DNA integrity testing) with the remaining 31 dogs.

Variable	Test	P value
Age	Mann-Whitney U	0.840
Body weight	Mann-Whitney U	0.830
Fresh volume	Mann-Whitney U	0.702
Concentration	Mann-Whitney U	0.229
TSO	Mann-Whitney U	0.365
Fresh TM	Mann-Whitney U	0.440
Fresh PM	Mann-Whitney U	0.537
Fresh VCL	Mann-Whitney U	0.856
Fresh VSL	Mann-Whitney U	0.582
Fresh VAP	Mann-Whitney U	0.314
Fresh LIN	Mann-Whitney U	0.452
Fresh STR	Mann-Whitney U	0.390
Fresh WOB	Mann-Whitney U	0.242
Fresh ALH	Mann-Whitney U	0.497
Fresh BCF	Mann-Whitney U	0.049
Fresh normal morphology	Mann-Whitney U	0.265
VEGF	Mann-Whitney U	0.195
Size class (breed proxy)	Chi-square	0.696

Table S2. Spearman correlation coefficients (ϱ) between blood plasma VEGF concentration, testicular artery Doppler parameters, and the post-thaw drop in plasma membrane integrity, acrosome integrity, mitochondrial mem-brane potential, and DNA integrity (n = 33); VEGF, vascular endothelial growth factor, peak systolic velocity (PSV), end-diastolic velocity (EDV), pulsatility index (PI), resistive index (RI), and systolic/diastolic ratio (S/D)

Variable	Drop in membrane integrity (ϱ / p)	Drop in acrosome integrity (ϱ / p)	Drop in mitochondrial membrane potential (ϱ / p)	Drop in DNA integrity (ϱ / p)
VEGF	ϱ = 0.299, p = 0.091	ϱ = -0.287, p = 0.106	ϱ = 0.031, p = 0.862	ϱ = 0.148, p = 0.411
PSV	ϱ = -0.075, p = 0.677	ϱ = -0.158, p = 0.381	ϱ = -0.202, p = 0.260	ϱ = 0.250, p = 0.160
EDV	ϱ = -0.095, p = 0.600	ϱ = 0.152, p = 0.397	ϱ = 0.063, p = 0.729	ϱ = 0.073, p = 0.688
PI	ϱ = -0.039, p = 0.830	ϱ = -0.258, p = 0.147	ϱ = -0.294, p = 0.097	ϱ = -0.053, p = 0.768
RI	ϱ = -0.051, p = 0.779	ϱ = -0.266, p = 0.134	ϱ = -0.305, p = 0.085	ϱ = -0.036, p = 0.844
S/D	ϱ = -0.016, p = 0.932	ϱ = -0.240, p = 0.178	ϱ = -0.281, p = 0.113	ϱ = -0.023, p = 0.900
